# Musculoskeletal disorders in padel: from biomechanics to sonography

**DOI:** 10.1007/s40477-023-00869-2

**Published:** 2024-04-05

**Authors:** Giulio Cocco, Vincenzo Ricci, Antonio Corvino, Michele Abate, Adele Vaccaro, Carlotta Bernabei, Vito Cantisani, Gianfranco Vallone, Corrado Caiazzo, Massimo Caulo, Andrea Delli Pizzi

**Affiliations:** 1 Department of Neuroscience, Imaging and Clinical Sciences, “G. D’Annunzio” University, 6610 Chieti, Italy; 2 Unit of Ultrasound in Internal Medicine, Department of Medicine and Aging Sciences, University “G. D’Annunzio”, Chieti, Italy; 3 Physical and Rehabilitation Medicine Unit, Luigi Sacco University Hospital, ASST Fatebenefratelli-Sacco, 20157 Milan, Italy; 4 Medical, Movement and Wellbeing Sciences Department, University of Naples “Parthenope”, Via Medina 40, 80133 Naples, Italy; 5 IRCSS Ospedale San Raffaele, Via Olgettina 60, 20132 Milan, Italy; 6 Department of Radiology, SS. Annunziata Hospital of Chieti, University “G. D’Annunzio”, Chieti, Italy; 7 Department of Radiology, Oncology, Sapienza-University of Rome, Anatomo-Pathology, Rome, Italy; 8 Department of Medicine and Health Sciences “V. Tiberio”, University of Molise, 86100 Campobasso, Italy; 9 Department of Innovative Technologies in Medicine and Dentistry, University “G. D’Annunzio”, Chieti, Italy

**Keywords:** Ultrasound, Padel, Biomechanics, Musculoskeletal, Injuries

## Abstract

Padel is a racket sport, combining high-frequency and low-intensity athletic gestures, that has been gaining growing scientific interest in recent years. Musculoskeletal injuries are very common among padel players with an incidence rate of 3 per 1000 h of training and 8 per 1000 matches. To the best of our knowledge, a comprehensive collection describing the most common sonographic findings in padel players with musculoskeletal injuries is lacking in the pertinent literature. In this sense, starting from the biomechanical features of padel-specific gestures we have reported the ultrasonographic patterns of most frequent injuries involving the upper limb, the trunk, and the lower limb. Indeed, comprehensive knowledge of the biomechanical and clinical features of musculoskeletal injuries in padel is paramount to accurately perform a detailed ultrasound examination of the affected anatomical site. So, the present investigation aims to provide a practical guide, simple and ready-to-use in daily practice, to optimize the sonographic assessment of padel players by combining it with the clinical findings and the biomechanical features of athletic gestures.

## Introduction

Padel is a racket sport following rules and a scoring system similar to tennis. Enclosed synthetic glass and metal court with a size of 20 m × 10 m, allowing the ball to rebound off the lateral and back walls can be considered the main difference with the tennis. This peculiar court with perimetral walls results in specific gestures such as fast-paced lateral movements, quick changes in direction, a high number of overhead shots, and explosive movements toward the net [1].

Musculoskeletal injuries are very common among padel players with an incidence rate of 3 per 1000 h of training and 8 per 1000 matches [2]. A systematic review published by Dahmen et al. in 2023 involving 8 studies with 2022 participants defined an overall prevalence range of injuries in padel of 40%-90%, with the elbow identified as the most frequent anatomical site [2]. In the same year, Tagliafico et al., confirmed the lateral elbow tendinopathy as the most frequent pathological condition affecting the upper limb in padel players through a survey involving 800 subjects [3]. Interestingly, the same authors reported the majority of musculoskeletal injuries were located on the lower limb (*N* = 49/85), most of which affected the knee joint (*N* = 16/49) [3].

Priego Quesada et al., in 2018 recruited 80 non-professional padel players and using a self-administered questionnaire described ankle sprain as the most common musculoskeletal injury; and, subacromial-subdeltoid bursitis and lateral epicondylitis as the most frequent pathologies affecting the upper limb [4].

Pérez et al., in 2023 published a retrospective cross-sectional study involving 44 musculoskeletal injuries of 36 players who participated in the World Padel Tour 2021 reporting the muscle injuries of the lower leg as the most common (28.13%) [5]. The same research group also described a higher prevalence of muscle injuries in top-ranked padel players and, tendon injuries in low-ranked players [5].

Based on the aforementioned epidemiological data described above, the present investigation aims to accurately describe common sonographic findings characterizing the musculoskeletal injuries in padel players, starting by the biomechanical features of their specific gestures. Indeed, a comprehensive knowledge of padel-specific movements can be considered paramount in order to perform a detailed sonographic assessment of the affected anatomical site. The upper limb, trunk, and lower limb have been identified as the three main sections of the manuscript. For each of them, biomechanical features of specific gestures and movements have been described and matched with sonographic findings that commonly characterize the corresponding musculoskeletal injuries.

We strongly believe the present research can be considered a useful and practical guide to accurately perform the ultrasound (US) examination in padel players in daily practice.

## Upper limb

### Shoulder

#### Biomechanics

The biomechanical function of the subacromial-subdeltoid (SASD) bursa is to protect the underlying tendon tissue from wear by dissipating friction in the anatomical space between the rotator cuff and the overlying coracoacromial arch. The latter can be considered a fat-filled anatomical interface in which the synovial layers of the SASD bursa glide over each other [6].

**Fig. 1:**
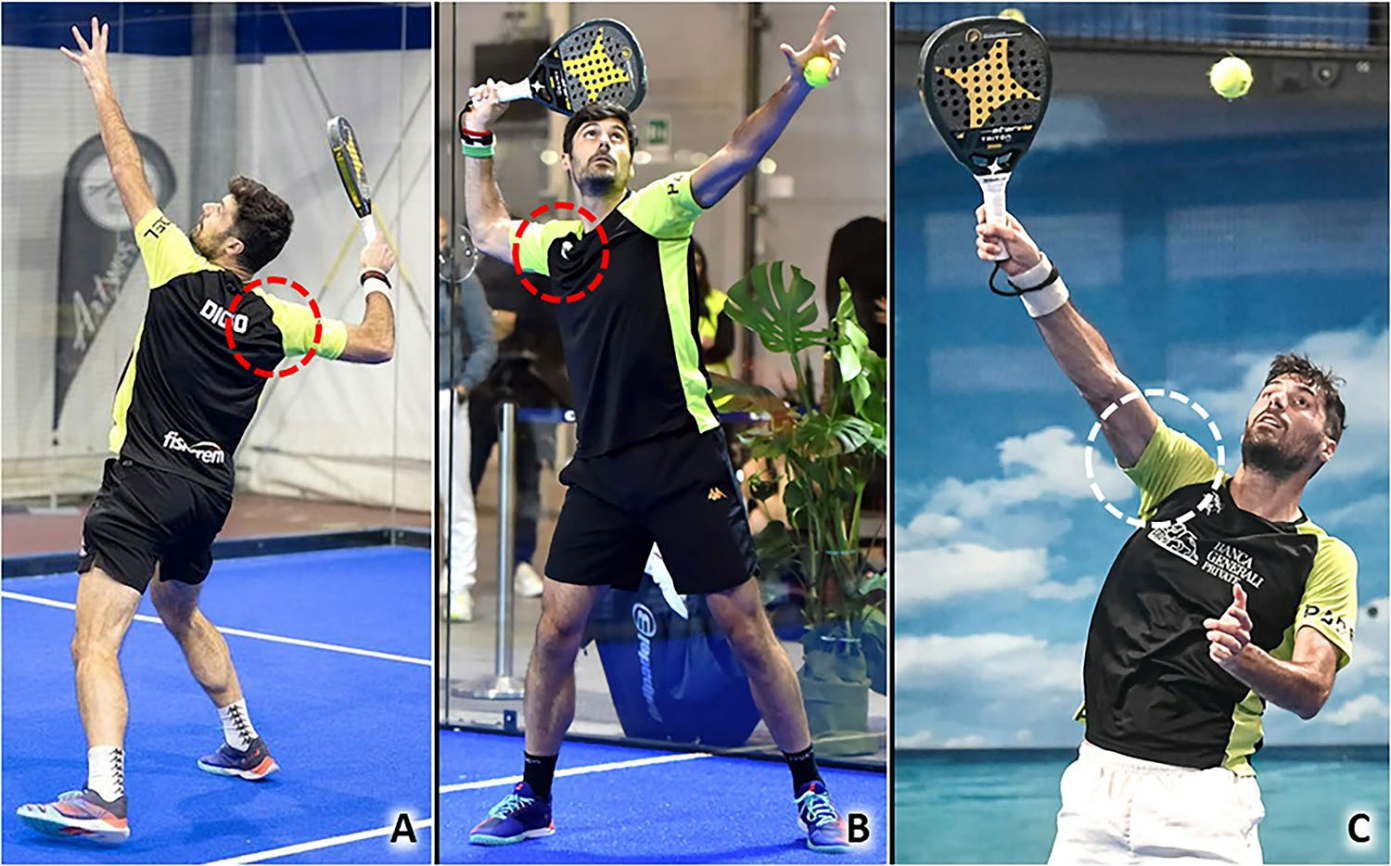
Combined movements of abduction (over 90 degrees) and external rotation *(red dotted circle)* of the shoulder (**A, B**), and elevations *(white dotted circle)* of the upper limb overhead (**C**), are very frequently performed during a padel match considering the court’s size and, the upward trajectory of the ball due to the multiple bounces on the perimetral walls

Anterosuperior impingement syndrome is a common cause of SASD bursitis in racquet sports. Indeed, overhead activities with repetitive movements of abduction and elevation of the humerus over 90 degrees reduce the acromion-humeral space with potential impingement of the rotator cuff and synovial bursa (Table 1) [7]. As previously mentioned, padel is played on a smaller court than tennis, leading to an increase in the frequency of overhead shots taken and the number of combined movements of abduction-external rotation of the shoulder to perform them (Fig. 1) [4]. Among many types of overhead strokes, smash (classic, par 3, and par 4), rulo or roll, vibora, bandeja, cuchilla, gancho and chancletazo are the most frequent. The latter, also known as “a hit with a big flip-flop”, is an aggressive, flat, finishing shot from a player very close to the net when they receive an easy ball to their forehand side. Moreover, the frequent bounces on the perimetral walls cause the ball to rise upwards, away from the ground, several times during a single match favoring the execution of multiple overhead shots. Muñoz et al., in 2022 identified the shoulder as the second most common anatomical site of injury of the upper body, after the elbow, in a cohort of 950 amateur padel players using a specific questionnaire [8].

**Table 1:** From Biomechanics to Sonography

Pathology	Biomechanics	Sonography
SASD bursitis	Repetitive elevations of the upper limb and repeated ABER movements of the shoulder	Bursal effusion, thickening of the synovial walls^a^ ± hypervascularization
Lateral elbow tendinopathy	Repeated contractions of the forearm extensor muscles, repetitive elongations and eccentric loads of the CET, rubbing of the CET with the underlying humeral capitellum	Hypoechoic thickening of the tendon ± hypervascularization, focal injury of the tendon, intra-tendinous calcifications, bony spur of the LE/ME, abnormalities of tendon-bone interface
Medial elbow tendinopathy	Explosive combinations of wrist flexion and forearm pronation, numerous topspin and slice spin	
Wrist tendinopathies	Repetitive ulnar and radial deviations of the wrist, rapid torsional movements of the wrist	Tendon sheath effusion, tenosynovial hypertrophy ± hypervascularization, retinacular thickening, tendinosis, focal injury of tendon tissue
Abdominal wall muscles injuries	Explosive combination of flexion and rotation of the trunk to force the ball during the impact phase of the overhead strokes	Disruption of muscle fibers, hematoma, fascial discontinuity, muscular stump retraction, perilesional hypervascularization
Patellar tendinopathy	Valgus/rotatory stresses on the knee, eccentric load in the landing phase of the jumps	Hypoechoic thickening of the tendon ± hypervascularization, focal injury of the tendon, calcifications, abnormalities of tendon-bone interface
MGM injuries	Repetitive eccentric contractions to rapidly change direction and perform jump shots	Muscle injury, anterior aponeurosis interruption, free aponeurosis discontinuity, hematoma in the inter aponeurotic space
Achilles tendinopathy	Repetitive elongations of the tendon, repeated concentric and eccentric loads of the tendon, overstretch of the crural fascia with mechanical irritation of the Achilles paratenon	Hypoechoic thickening of the tendon ± vascular signals, hypoechoic thickening of the paratenon ± neo-vessels, partial tear of the tendon, calcifications, superficial/deep retrocalcaneal bursitis, abnormalities of the tendon-bone interface

*SASD* subacromial-subdeltoid, *ABER* abduction + external rotation, *CET* common extensor tendon, *LE* lateral epicondyle, *ME* medial epicondyle, *MGM* medial gastrocnemius muscle

^a^E.g., nodular thickening, hypertrophic synovial villi

Usually, de-coaptation exercises (e.g., Codman’s pendulum) and strengthening for the humeral head depressor muscles are performed during athletic training by professional padel players to rebalance the force vectors involved in the vertical shoulder movements [9].

#### Clinical findings

The clinical scenario of SASD bursitis often presents with anterolateral pain of the shoulder exacerbating over 90 degrees of abduction. Padel players with chronic nodular bursitis may also complain of a feeling of painful clicking during the elevation of the upper limb due to the snap of thickened synovial walls of the SASD bursa under the coracoacromial arch [6]. Of note, the snap usually occurs in the intermediate degrees of the shoulder range of motion. On physical examination, the patient commonly presents multiple points of tenderness over the anterior and lateral surface of the deltoid region and a positive painful arch test during active abduction of the shoulder [9].

#### Sonographic findings

**Fig. 2:**
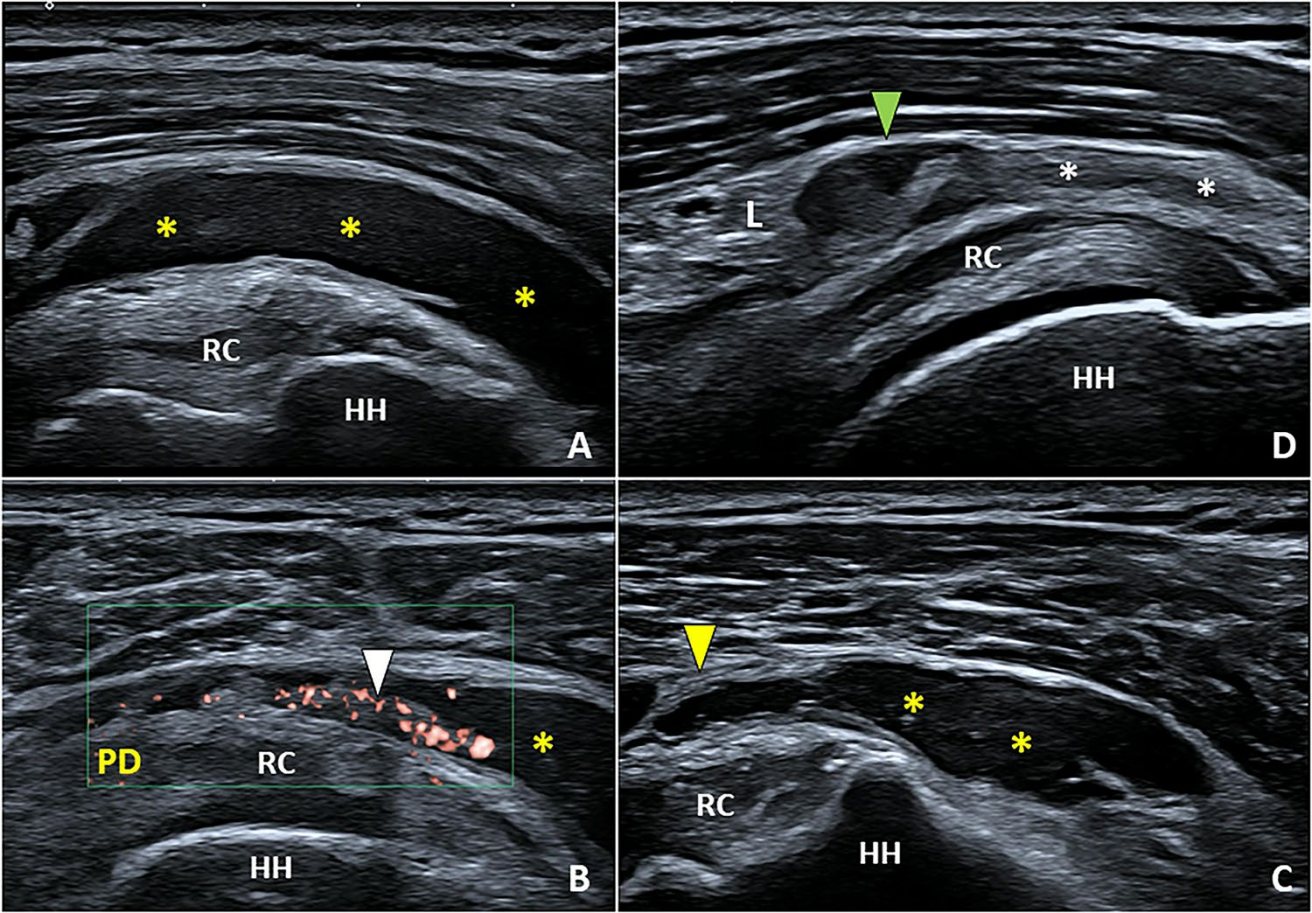
In the acute phase, the effusion *(yellow asterisks)* is the most common sonographic finding defining exudative bursitis (**A**). Progressively, synovial hypertrophy (**B**) with hypervascularization *(white arrowhead)* and fibrotic septae *(yellow arrowhead)* can develop within the bursal cavity (**C**). Adhesive bursopathy (**D**) shows a well-defined, hypoechoic, nodular thickening *(green arrowhead)* of the synovial tissue *(white asterisk)* close to the overlying hyperechoic coracoacromial ligament *(L)*. *HH* humeral head, *RC* rotator cuff, *PD* power Doppler

Anechoic effusion inside the lumen of the SASD bursa is the most common sonographic finding in cases of acute and sub-acute exudative bursitis (Fig. 2). Depending on the position of the patient during the US examination, the effusion may distribute mainly in the lateral portion of the bursa (tear sign) or in its medial portion at the level of the coracoid space mimicking an articular effusion involving the subscapular recess of the glenohumeral joint [10]. In the chronic phase of anterolateral shoulder impingement, a hypoechoic proliferation of the synovial layers can be observed floating within the effusion – i.e., hypertrophic bursitis [9]. Accurately setting the color/power Doppler by adjusting the size/position of the box and the pulse repetition frequency, hypervascularization can be sometimes depicted within the hypertrophic synovial tissue of the bursa (Fig. 2) [11].

A third sonographic pattern of SASD bursitis is known as adhesive bursopathy, referring to a thickening of the bursal walls greater than 3 mm with or without nodular formations (Fig. 2) [6]. In the authors’ experience, this type of SASD bursitis is the most challenging to diagnose especially for beginners considering the absence of effusion that acts as a natural contrast agent to easily visualize the bursal walls. Unlike the hypertrophic type, adhesive bursopathy usually does not show hyperemia at color/power Doppler.

Lastly, a dynamic ultrasound assessment can be performed to promptly visualize the eventual snapping or pinching of the SASD bursa under the coracoacromial arch [12].

### Elbow

#### Lateral elbow tendinopathy

##### Biomechanics

**Fig. 3:**
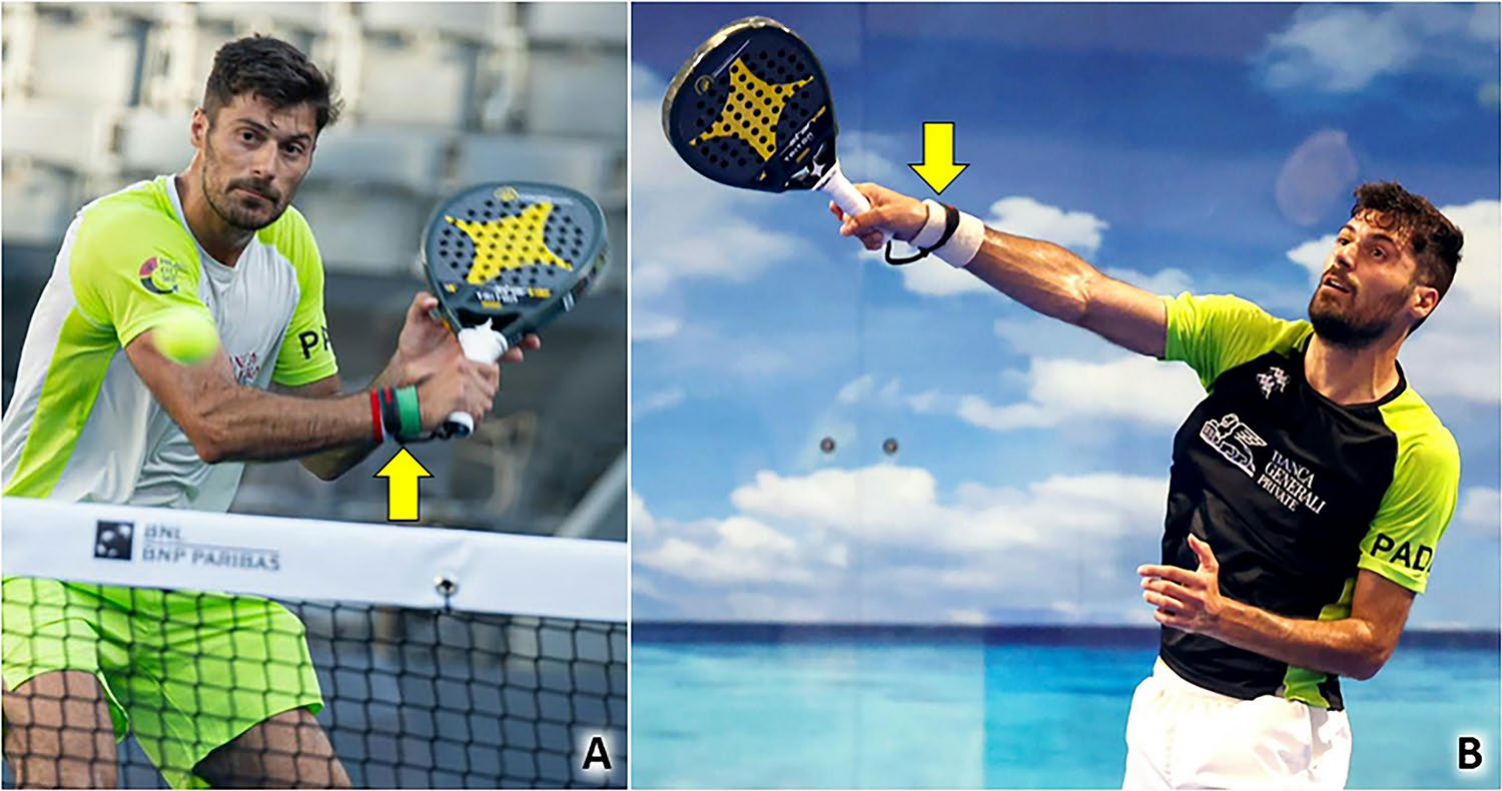
An expert padel player starts and keeps the one-hand backhand stroke with the extended wrist *(yellow arrow)* to reduce the eccentric load on the common extensor tendon in the lateral compartment of the elbow both in the pre-impact phase (**A**) and after the impact of the ball (**B**)

Lateral elbow tendinopathy, also known as lateral epicondylitis or tennis elbow, typically involves the extensor carpi radialis brevis (ECRB) tendon due to microtrauma from repetitive backhand strokes, particularly with the one-handed technique [2]. Of note, expert and novice players use different techniques to perform backhand strokes. Expert players hit the ball with a hyperextended wrist and keep the wrist extended throughout impact (Fig. 3); instead, novice players strike the ball with a flexed wrist and, move the wrist into further flexion throughout impact leading the common extensor tendon (CET) of the elbow to progressive eccentric stress [13]. For this reason, novice players are more prone to lateral elbow tendinopathy if compared to expert players.

The repeated contraction of the forearm extensor muscles and the corresponding mechanical overload of the proximal origin of the ECRB tendon over the lateral epicondyle (LE) results in micro-tearing of the tendon tissue with subsequent failure of the healing process and angiofibroblastic degeneration (i.e., tendinosis) [14].

Other biomechanical aspects representing potential risk factors of lateral tendinopathy in padel players are the repetitive varus stresses of the elbow; and, the continuous rubbing of the ECRB tendon against the lateral edge of the capitellum, coupled with its compression between the extensor carpi radialis longus muscle and the underlying bone, during the extension phase of the elbow or to perform specific gestures and strokes such as the backhand or the backhand volley (Table 1) [15].

Lastly, the high number of vibrations, originating from the padel racket at the ball contact due to hard materials, seems to be proximally transmitted to the CET increasing the risk of tendinopathy. In this sense, a soft racket core and anti-vibration systems should be considered to optimize its technical features. Indeed, the density of core materials (rubber core, foam core, hybrid core, etc.) highly influences the control and power of the racket [16].

##### Clinical findings

Pain or burning around the LE, often radiating along the dorsal surface forearm, are the most common symptoms complained by the athlete. Both are usually triggered or exacerbated by active contraction of the common extensor mass of the forearm which puts tension on the corresponding tendon at the elbow. Weakness in gripping may also be referred by the patient. Severe tenderness by palpating the origin of the ECRB tendon can be considered the most common clinical finding during physical examination. Cozen’s test, Maudsley’s test, and forearm supination with the elbow in the extended position are clinical tests aiming to reproduce lateral elbow pain [17].

Direct compression of the posterolateral surface of the radial head, coupled with passive pronation/supination of the forearm, can be performed to test the eventual presence of radiohumeral synovitis and/or radial head chondropathy mimicking the lateral elbow tendinopathy [18].

##### Sonographic findings

**Fig. 4:**
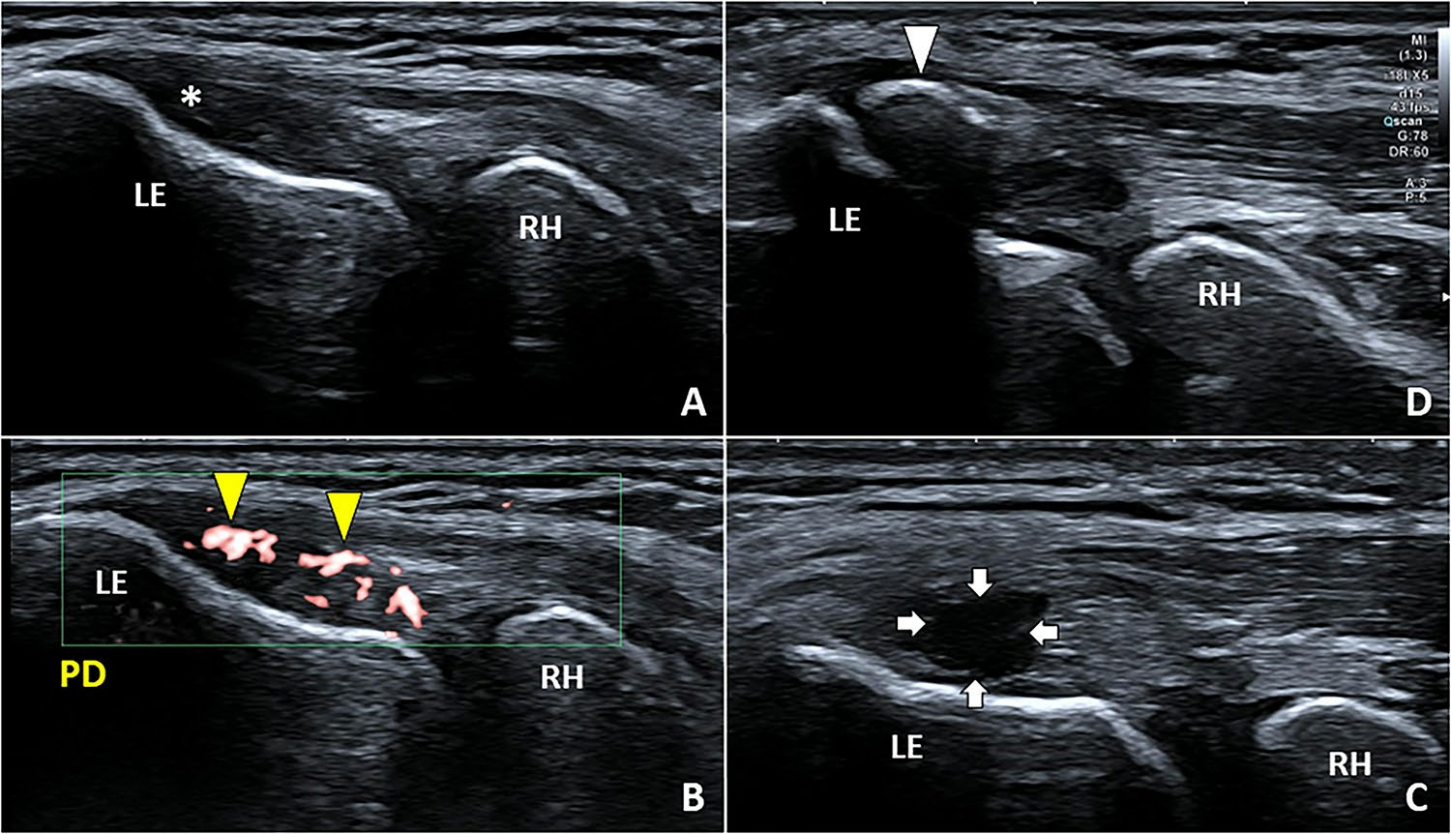
Hypoechoic thickening *(white asterisk)* of the CET in B-Mode (**A**) and neo-vessels *(yellow arrowheads)* in color/power Doppler (**B**) are the most common sonographic findings in padel players with lateral elbow tendinopathy. Focal injury *(white arrows)* of the CET (**C**) and calcific depositions *(white arrowheads)* can also be identified especially in players with recalcitrant lateral elbow pain (**D**). *LE* lateral epicondyle, *RH* radial head, *PD* power Doppler

Ultrasound assessment of the lateral elbow compartment is usually performed with the elbow flexed to 90 degrees and the forearm pronated. Angiofibroblastic hyperplasia, also known as tendinosis, is the most common sonographic finding in padel players with lateral elbow pain. Hypoechoic thickening of the CET and disappearance of the normal fibrillar pattern in the longitudinal view is its typical appearance (Fig. 4) [14]. Combining the transverse plane with the longitudinal one the exact portion of the common tendinous mass affected by the degenerative changes can be accurately identified to plan a tailored rehabilitation approach [19]. Using color/power Doppler the vascular pattern of degenerated tendon tissue due to the local proliferation of neovessels can be assessed by accurately setting the size of the Doppler box, the gain, and the pulse repetition frequency [11]. Hypervascularization of the CET usually presents larger and horizontally oriented vessels (feeding vessels) running in the subcutaneous tissue and, smaller vertically oriented vessels (penetrating vessels) that creep into the tendon tissue [14].

Focal tendinosis of the CET can be coupled with partial injury of the tendon tissue, calcific deposits (Fig. 4), bony spur of the lateral epicondyle, and irregularities of the tendon-bone interface in padel players with lateral elbow pain [20–22]. The latter is also defined as mechanical enthesopathy and usually presents irregularities of the cortical bone of the lateral epicondyle, lamellar calcifications of the tendon collagen fibers, and hypervascularization of the hypoechoic cartilaginous plate of the tendon-bone interface [14, 23].

#### Medial elbow tendinopathy

##### Biomechanics

**Fig. 5:**
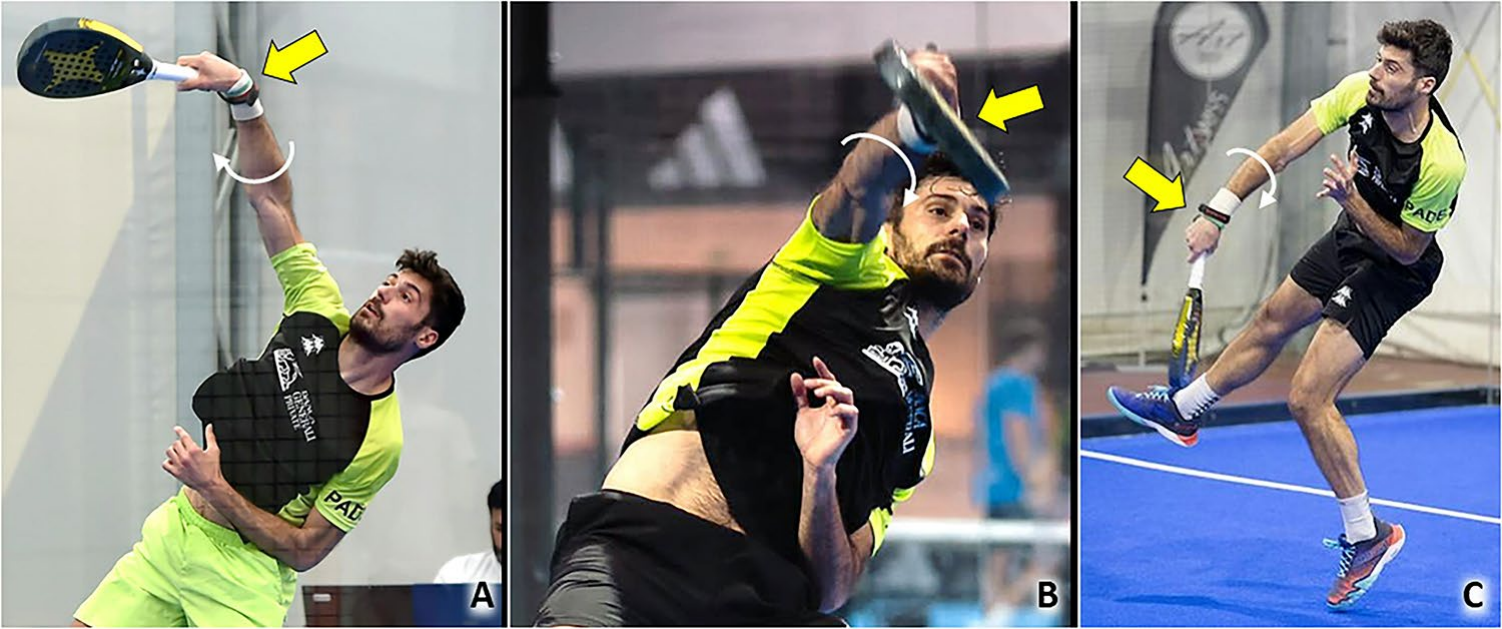
By rapidly activating the pronator teres and flexor carpi radialis muscles, a sudden flexion of the wrist *(yellow arrow)* coupled with complete pronation of the forearm *(white curved arrow)* can be performed by elite padel players—the “whipping” effect (**A**, **B**). The aforementioned gestures result in a global torsional movement of the upper limb pivotal to increase the power/velocity of the ball and control its trajectory (**C**)

Medial elbow tendinopathy is commonly known as golfer’s elbow. Degenerative changes of the common flexor-pronator tendon (CFPT) are usually related to the overload of the pronator teres and flexor carpi radialis muscles [24]. Considering the small size of the padel court, the number of shots from the halfway point of the court and at the net is much higher compared to other sports racquet as tennis (Table 1) [25]. These shots are often performed with an explosive combination of wrist flexion and forearm pronation to smash the ball to the ground with maximal power – the “whipping” effect such as in the smash, especially the par 3 smash (Fig. 5). Moreover, the global torsional movement of the upper limb resulting from the simultaneous wrist flexion and forearm pronation allows the athlete to optimize the trajectory of the ball targeting a specific segment of the opposite half of the court. In this sense, unlike lateral elbow tendinopathy, medial tendinopathy is more common in professional padel players rather than in novice players due to the aforementioned specific gestures frequently and massively involving the flexor and pronator muscles of the forearm.

Moreover, rapid pronation of the forearm during the forehand stroke is performed by the padel player, by rolling the racket face over the ball via the area of contact, to generate a topspin effect on the ball. Likewise, by pronating the wrist on the backhand the slice spin effect is generated. In this sense, repetitive and explosive contractions of the pronator teres muscle are pivotal to performing shots that put the opponent in difficulty. Lastly, some padel-specific shots such as the vibora and the bandeja – as well as the forehand and forehand volley—may lead to excessive mechanical stresses of the CFPT of the elbow.

##### Clinical findings

Medial elbow tendinopathy is characterized by pain of insidious onset, which is worsened by resisted forearm pronation and wrist flexion. Tenderness to palpation usually occurs over the medial epicondyle and, distally along the muscular belly of pronator teres and the flexor carpi radialis [26]. The authors strongly suggest also performing a 30-degree valgus stress test of the elbow to assess the ulnar collateral ligament that in pathological conditions can be considered the “big mime” of medial tendinopathy [26].

##### Sonographic findings

**Fig. 6:**
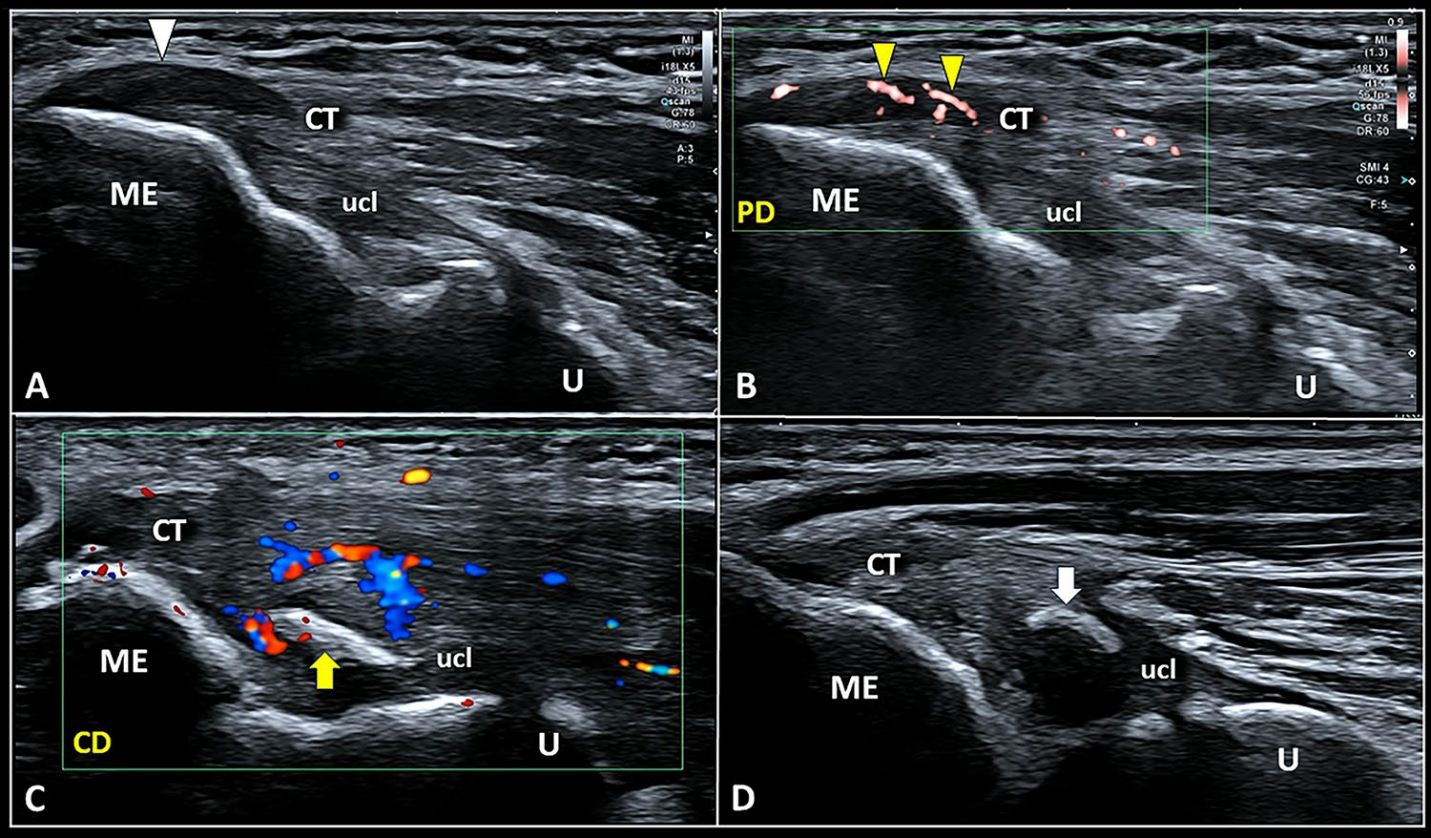
Hypoechoic thickening *(white arrowhead)* of the conjoint tendon *(CT)* in B-Mode (**A**) with neo-vessels *(yellow arrowheads)* penetrating the degenerated tendon tissue in power Doppler *(PD)* (**B**) are common sonographic findings in padel players with medial elbow pain. More rarely, a small bony fragment *(yellow arrow)* embedded in the ulnar collateral ligament *(ucl)* and surrounded by an intense hypervascularization (**C**) can be observed close to the painful medial epicondyle *(ME)*. Of note, a small ossicle *(white arrow)* within the collagen fibers of the ulnar collateral ligament *(ucl)*, commonly rounded in shape and not encircled by neo-vessels, can be identified also in asymptomatic volunteers (**D**). *U* ulna, *CD* color Doppler

The most common sonographic pattern in padel players with medial elbow pain is characterized by hypoechoic thickening of the CFPT with loss of fibrillar pattern (i.e., tendinosis) with or without intra-tendinous and peri-tendinous hypervascularization (Fig. 6). As previously mentioned for the CET, also the tendinosis of CFPT can be coupled with partial injury, calcific deposits, bony spur of the medial epicondyle, and enthesopathy of the tendon-bone junction [26, 27].

In padel players with recalcitrant medial elbow pain and poor response to conservative treatments focused on the tendon compartment, eventual sonographic abnormalities of the ulnar collateral ligament should be scrutinized. A small bony fragment linear-in-shape with hypervascularization of the surrounding soft tissues is a quite frequent pathological finding exactly located in the most painful site complained by the patient (Fig. 6). Of note, a dome-shaped ossicle can be normally identified within the ulnar collateral ligament of the elbow also in asymptomatic athletes (Fig. 6) and should not be misinterpreted as the pathological and painful bony fragment previously mentioned. In this sense, the different shape of the hyperechoic formation (linear vs. dome-shaped), and the abundant vascular signals surrounding the painful bony fragment but not the physiological ossicle can be considered a pivotal sonographic signs for the differential diagnosis.

### Wrist

#### Biomechanics

**Fig. 7:**
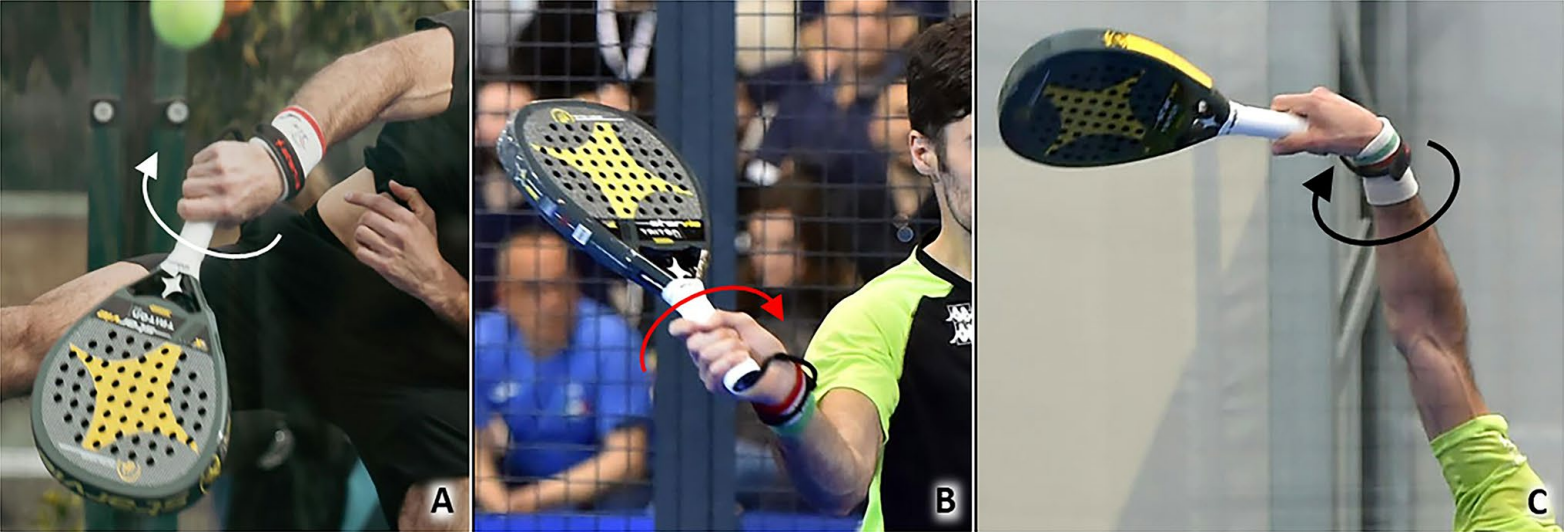
Repeated ulnar *(white curved arrow)* and radial *(red curved arrow)* deviations of the radiocarpal joint (**A**, **B**) and quick torsional movements *(black curved arrow)* of the wrist (**C**) during a padel match may progressively lead to pathologies of the extensor tendons

Repetitive ulnar and radial deviations of the wrist during the padel match to orientate the racquet and hit the ball may overload the wrist extensor tendons, especially the first and sixth compartments largely involved in these movements (Fig. 7). Likewise, rapid and repeated torsional movements of the wrist (“screwing” effect) to optimize the trajectory of the ball, produce topspin and slice spin effects, and force the stroke require violent contractions of the forearm muscles tensioning the corresponding tendons (Table 1).

Four are the traditional single-handed grip positions of the racket in padel: the continental, Eastern, semi-Western, and Western grips. Each hand grip position of the racket offers unique advantages and disadvantages, influencing the load to the wrist during gameplay. Interestingly, the Eastern grip, often preferred by expert padel players to increase the power of both forehand and backhand strokes and impart top-spin effects to the ball, particularly overloads the first extensor tendon compartment of the wrist – i.e., the abductor pollicis longus and extensor pollicis brevis [28]. Anatomically, the wrist extensor compartments are stabilized by an inverted U-shape fibrous retinaculum and, present a tenosynovial sheath surrounding the tendons to promote their smooth gliding. In this sense, tenosynovitis, tendinosis, and focal injury are the most common pathological changes involving the aforementioned tendons in padel players [29].

Lastly, repetitive flexion and extension of the wrist may also lead to extra frictions between the first and second (extensor carpi radialis brevis and extensor carpi radialis longus) compartment 4 cm proximally to the radiocarpal joint – i.e., the proximal intersection syndrome.

#### Clinical findings

Pain, focal swelling, and snapping are the most common clinical findings on the radial or ulnar side of the wrist related to the first and sixth tendon compartments respectively. Clinical tests by passively moving the wrist in a radial or ulnar direction (e.g., Finkelstein's maneuver) may provoke and/or exacerbate the aforementioned symptoms confirming the diagnosis. In chronic cases, synovial adhesions and retinacular hypertrophy can markedly impair the tendon gliding with a reduced active and passive wrist range of motion in different spatial planes.

#### Sonographic findings

**Fig. 8:**
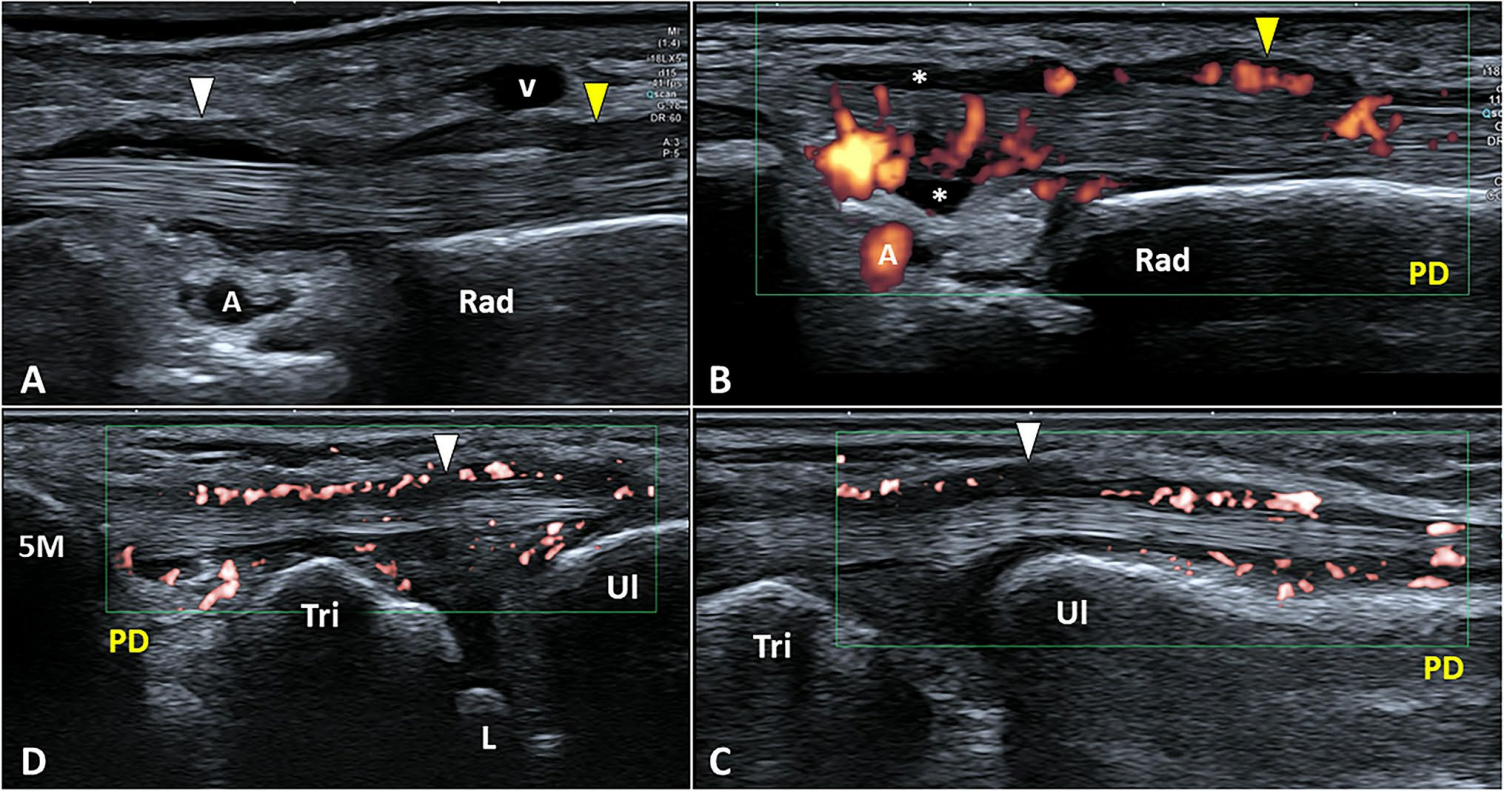
Thickening of the fibrous retinaculum *(yellow arrowhead)*, sheath effusion *(white asterisks)*, tenosynovial hypertrophy *(white arrowhead)* with hypervascularization of the first extensor compartment is visualized in a padel player with radial wrist pain (**A**, **B**). Likewise, tenosynovitis of the extensor carpi ulnaris tendon with multiple vascular signals within the hypertrophic synovial sheath *(white arrowhead)* can be observed on the ulnar side (**C**, **D**). *Rad* radius, *A* artery, *V* vein, *PD* power doppler, *Ul* ulna, *Tri* triquetrum, *L* lunate, *5M* fifth metacarpal bone

Tendon sheath effusion, tenosynovial hypertrophy, retinacular thickening, tendinosis, and partial injury of tendon tissue are the most common sonographic findings in wrist extensor tendons compartments of padel players (Fig. 8) [30, 31]. The presence of a vertical fibrous septum within the first compartment is often related to a “selective” synovitis involving the sheath of extensor pollicis brevis tendon and not the abductor pollicis longus tendon [32]. The color/power Doppler, especially in the acute phase, shows hypervascularization mainly involving the synovial tissue of the tendon sheath and, more rarely the tendon tissue itself.

Dynamic ultrasound assessment can be performed on padel players with feelings of painful clicks at the wrist. The most common sonographic findings are the instability of extensor carpi ulnaris tendon at the ulnar bony groove and, the snapping of the first extensor tendons compartment due to the entrapment within the thickened osteofibrous tunnel [33].

Aberrant vascular signals in the anatomical interface between the first and second extensor compartment, peri tendinous anechoic edema, and a painful sono-palpation of the intersectional area are the typical sonographic signs in athletes with proximal intersection syndrome of the wrist [34, 35]. Sometimes, local effusion can be confined inside a proper synovial neo-bursa located in between the tendons [36]. 

### Trunk

#### Biomechanics

**Fig. 9:**
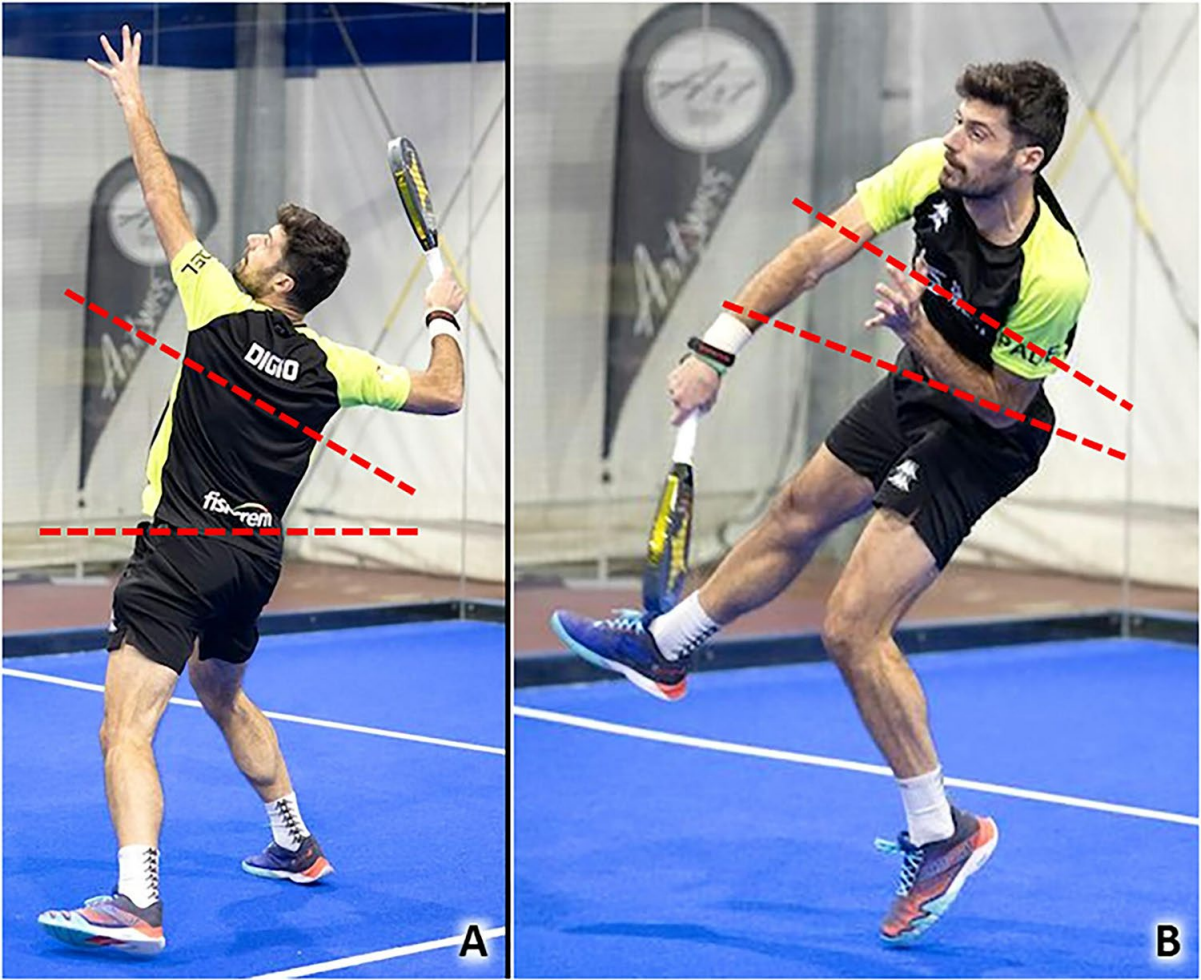
The transition from the loading to impact phase of overhead strokes during a Padel match requires a very rapid and explosive change of the shape of the core *(red dotted lines)*. Indeed, an eccentric contraction of abdominal muscles to control the hyperextension of the spine (**A**) is immediately followed by powerful concentric contractions to flex and rotate the trunk (**B**) transmitting the forces from the lower to upper limbs

Overhead strokes in padel require a great power of core muscles. Most of the overhead shots previously described are characterized by an early phase necessary to store the energy in the abdominal muscles; and, a late phase with rapid and violent release of the elastic energy to hit the ball as hard as possible (Fig. 9) [37]. The loading phase is characterized by hyperextension of the spine—with progressive elongation of the rectus abdominis muscles (eccentric contraction)—coupled with a twist of the trunk. In this way, elastic energy is stored in the trunk’s walls like a “spring mechanism”. Instead, during the impact phase, the abdominal muscles contract forcefully generating an abrupt flexion and rotation of the trunk to force the ball to the opposite side of the court with the maximal energy and velocity (Table 1). Indeed, the trunk acts as a functional bridge transmitting the forces from the lower limbs to the upper limbs. Especially in the acceleration phase of the trunk, the rectus and oblique abdominal muscles contralateral to the service side are exposed to a high risk of traumatic injury due to an explosive concentric contraction [37, 38].

#### Clinical findings

Pain, local ecchymosis, and loss of function/strength are the most common findings in patients with abdominal wall muscle injuries during the physical examination [38, 39]. For the rectus abdominis muscle, active contractions by flexing the thoracolumbar spine and/or its elongation during the spinal hyperextension can be considered additional clinical maneuvers to trigger local pain along the anterior surface of the abdominal wall. Likewise, active twisting of the trunk combined with lateral bending of the spine can be performed to test the oblique abdominal muscles [40]. The Valsalva maneuver can be used to increase the intra-abdominal pressure tensioning the corresponding abdominal wall muscles. In the authors’ experience, especially in low-energy trauma and small-size myofascial injuries, the clinical tests are not accurate enough to evoke pain and accurately localize the anatomical site of injury.

#### Sonographic findings

**Fig. 10:**
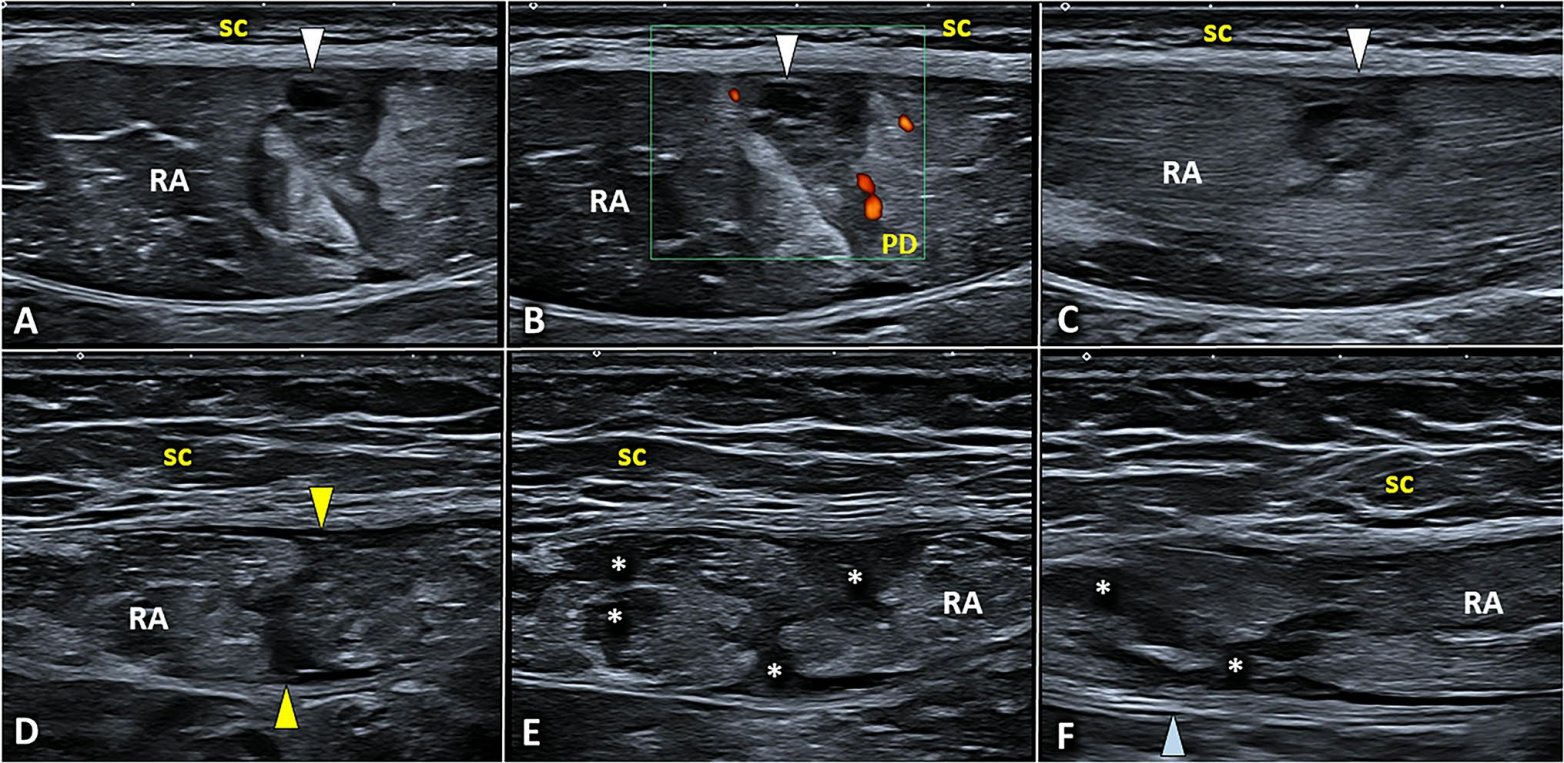
Combining transverse (**A**, **B**) and longitudinal (**C**) scans, a focal injury *(white arrowhead)* involving the superficial fibers of rectus abdominis muscle *(RA)*—with a mild peri-lesional hypervascularization (**B**) in power Doppler *(PD)*—can be observed in a professional padel player due to a powerful overhead stroke with massive contraction of the core muscles. Instead, a full-thickness tear *(yellow arrowheads)* of the rectus abdominis *(RA)* with blood collections *(white asterisks)* within the muscle belly can be visualized in transverse sonograms of an amatorial padel player (**D**, **E**). Of note, the longitudinal scan (**F**) confirms the anatomical integrity of the deep lamina of its fascial sheath *(blue arrowhead)*. *sc* subcutaneous tissue

The most common US findings are myofascial injuries due to indirect trauma (Fig. 10) [38]. A sonographic classification commonly used in clinical practice defines three main grades of severity for myofascial injuries after indirect trauma [41]: *Grade 1:* US examination may be either negative or exhibit focal/diffuse areas of increased echogenicity of the muscle tissue. The latter are related to the local edema and/or a micro-bleeding infiltrating the muscle tissue. *Grade 2:* US imaging shows areas of muscle fiber disruption with blood collections. Of note, in grade 2 the injury does not involve the entire cross-sectional area of the target muscle but only a part of it (partial tear). *Grade 3:* complete discontinuity or disruption of the target muscle with retraction of the corresponding muscle fibers (full-thickness tear).

In some athletes, and especially in the early phases after the trauma, the hyperechogenicity due to the acute bleeding can make the diagnosis of muscle injury quite challenging. In this sense, dynamic ultrasound examination with passive elongation and/or active contraction of the target muscle can be considered a powerful diagnostic tool to “open the gap” and promptly confirm the disruption of muscle tissue [42].

During the sonographic assessment of the rectus abdominis muscle, the structural integrity of its connective (hyperechoic) sheath should be accurately checked. Indeed, especially the deep lamina of the muscular sheath is widely crossed by perforating branches of the epigastric vessels, and; after a sport-related trauma, a rectus sheath hematoma should be considered among the potential complications [39].

**Fig. 11:**
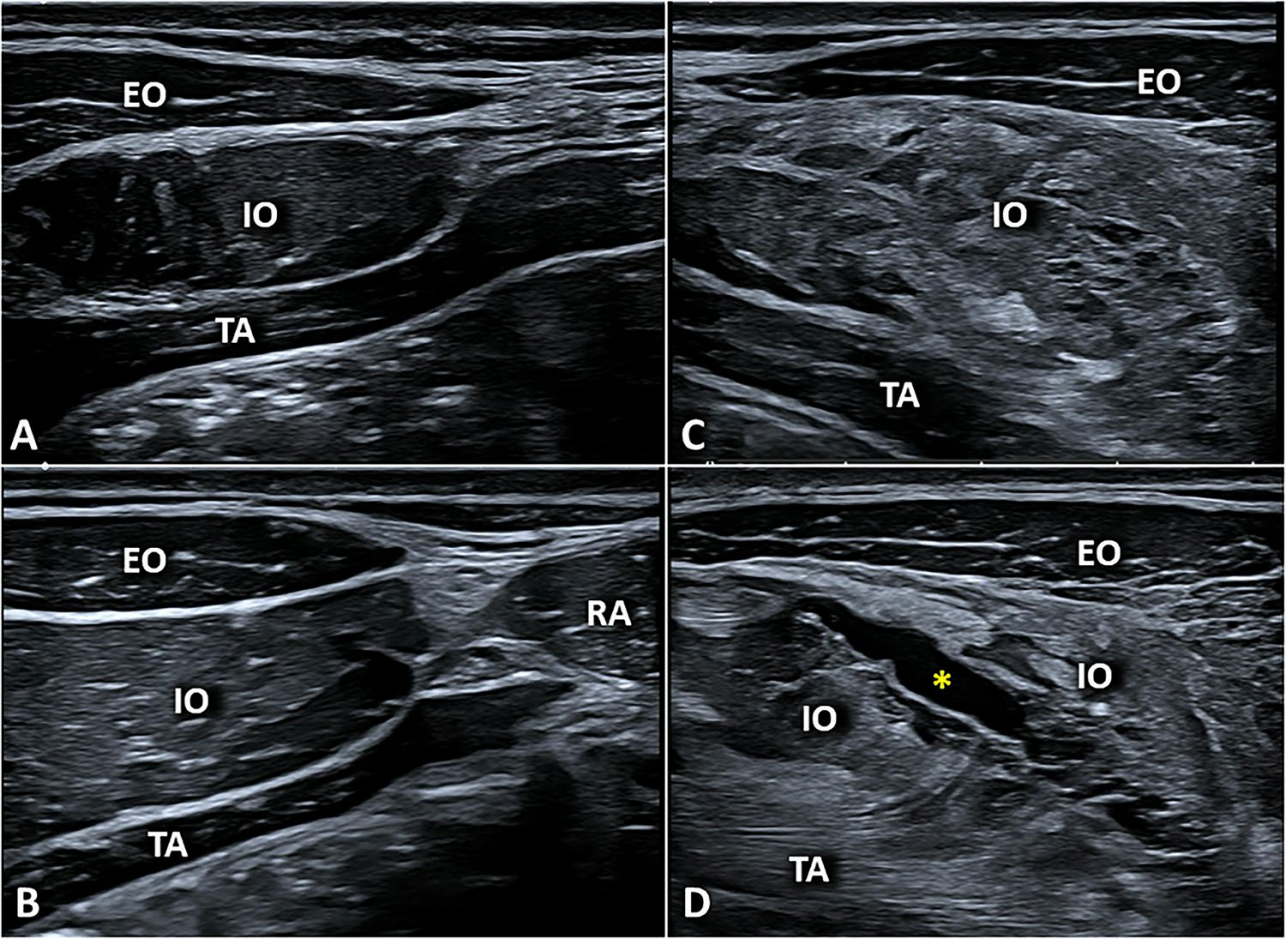
Comparative ultrasound scanning, in a transverse plane, of the normal (**A**, **B**) and painful (**C**) side of the anterolateral abdominal wall clearly shows a massive thickening and hyperechogenicity of the left internal oblique muscle *(IO)* due to severe intramuscular edema with a loss of the normal echotexture. Shifting the probe distally (**D**), disruption of its muscle fibers with a large intramuscular hematoma *(yellow asterisk)* has been identified confirming the post-traumatic myofascial injury with a sparing of the overlying external oblique muscle *(EO)*. *TA* transversus abdominis muscle, *RA* rectus abdominis muscle

Shifting the probe laterally to the rectus abdominis muscle the external oblique, internal oblique, and transverse abdominis muscles can be visualized. The internal oblique muscle can be injured during an extreme and unbalanced eccentric contraction to twist the trunk—the so-called “side strain syndrome” (Fig. 11) [43].

## Lower limb

### Knee

#### Biomechanics

**Fig. 12:**
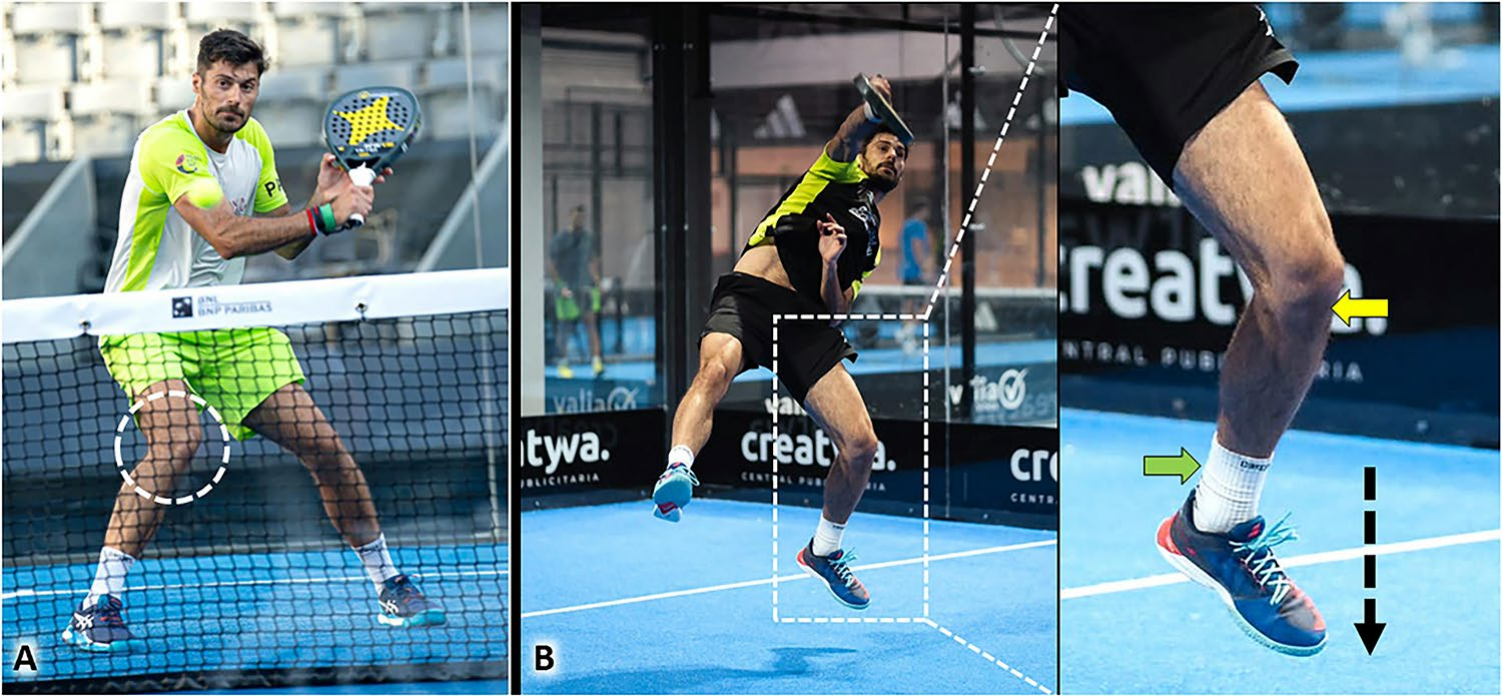
Rapid combined knee flexion-rotation movements *(white dotted circle)* are often necessary to correctly orientate the trunk and hit the ball at the optimal height (**A**). The patellar tendon *(yellow arrow)* and Achilles tendon *(green arrow)* function as shock absorber cords during the landing phase of the jump *(black dotted arrow)* modulating the velocity of braking and reducing the risk of knee/ankle sprain (**B**)

Considering the small size of the court and the presence of perimeter walls involving vertical bounces, the trajectories of the ball during a padel match lead the athlete to perform extremely rapid changes of direction and abrupt braking to reach it [1]. So, the knee extensor mechanism mainly composed of the quadriceps muscle and tendon, the patella, and the patellar tendon (PT) is pivotal to dynamically stabilizing the knee during the multiple valgus/rotatory stresses (Table 1). Moreover, the landing phase of jumps involves an eccentric load on the PT on the knee and the Achilles tendon at the ankle to accurately control the braking of the lower limb on the ground (Fig. 12).

#### Clinical findings

Anterior knee pain, mainly localized at the inferior pole of the patella, is the main clinical finding complained by the padel player with patellar tendinopathy. In the early phase, the pain is triggered by intense workouts or specific sports gestures; but, progressively if not properly managed, it can present also at rest or during common movements such as rising from a chair or climbing the stairs [44]. The most painful site on palpation during physical examination is the inferior edge of the patella where the proximal segment of PT is inserted. The maximal flexion can evoke anterior knee pain putting in tension the PT and mechanically stressing its enthesis to the inferior pole of the patella. Lastly, the athlete may be asked to perform a functional test as the squat – at different degrees of knee flexion—which can evoke pain in both the eccentric and concentric phases of the exercise.

#### Sonographic findings

**Fig. 13:**
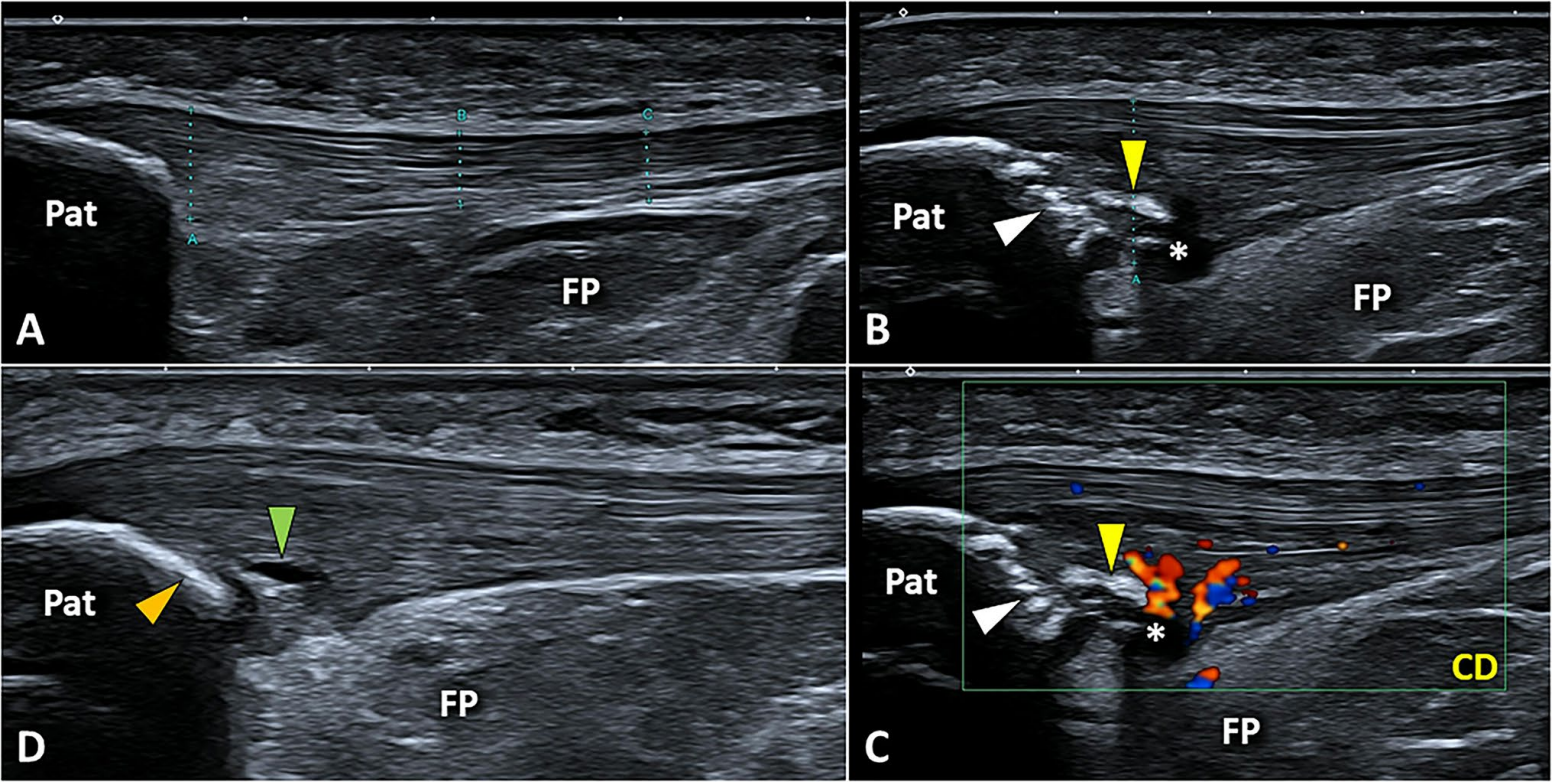
Normally (**A**), the patellar tendon presents a fibrillar echotexture in the longitudinal scan; instead, a hypoechoic thickening of the deep and proximal fibers *(white asterisk)*, lamellar calcifications *(yellow arrowhead)*, and cortical irregularities *(white arrowhead)* of the inferior pole of the patella *(Pat)* can be commonly observed in padel players with patellar tendinopathy (**B**). The color Doppler *(CD)* depicts neo-vessels (**C**) originating from the Hoffa fat pad *(FP)* and infiltrating the hypoechoic degenerated tendon tissue *(white asterisk)*. More rarely, an isolated longitudinal tear of the PT *(green arrowhead)* can be identified with a regular enthesis *(orange arrowhead)* (**D**)

The most common sonographic finding in padel players with patellar tendinopathy is the hypoechoic thickening of the inferior and proximal fibers of PT with loss of the fibrillar pattern. Intratendinous calcific deposits and abnormalities of the tendon-bone interface are commonly associated with the aforementioned sonographic signs of tendinosis (Fig. 13). The latter sonographic sign, clinically known as mechanical enthesopathy, is usually characterized by the pitting of the cortical bone of the inferior pole of the patella and thin linear in shape enthesophytes [45]. In doubtful cases, dynamic sonographic assessment with active extension of the knee can be performed to optimize the differential diagnosis between the focal tendinosis and the partial tear of the deep fibers of PT [46].

Using the color/power Doppler hypervascularization can be observed with neo-vessels usually originating from the underlying Hoffa fat pad and invading the degenerated tendon tissue. Indeed, in the pertinent literature, the US-guided disruption of the neo-vessels/neo-nerves crossing the tendon-fat pad interface has been proposed as an interventional technique to manage chronic recalcitrant patellar tendinopathy (i.e., the US-guided scraping of PT) [47].

### Posterior leg

#### Biomechanics

**Fig. 14:**
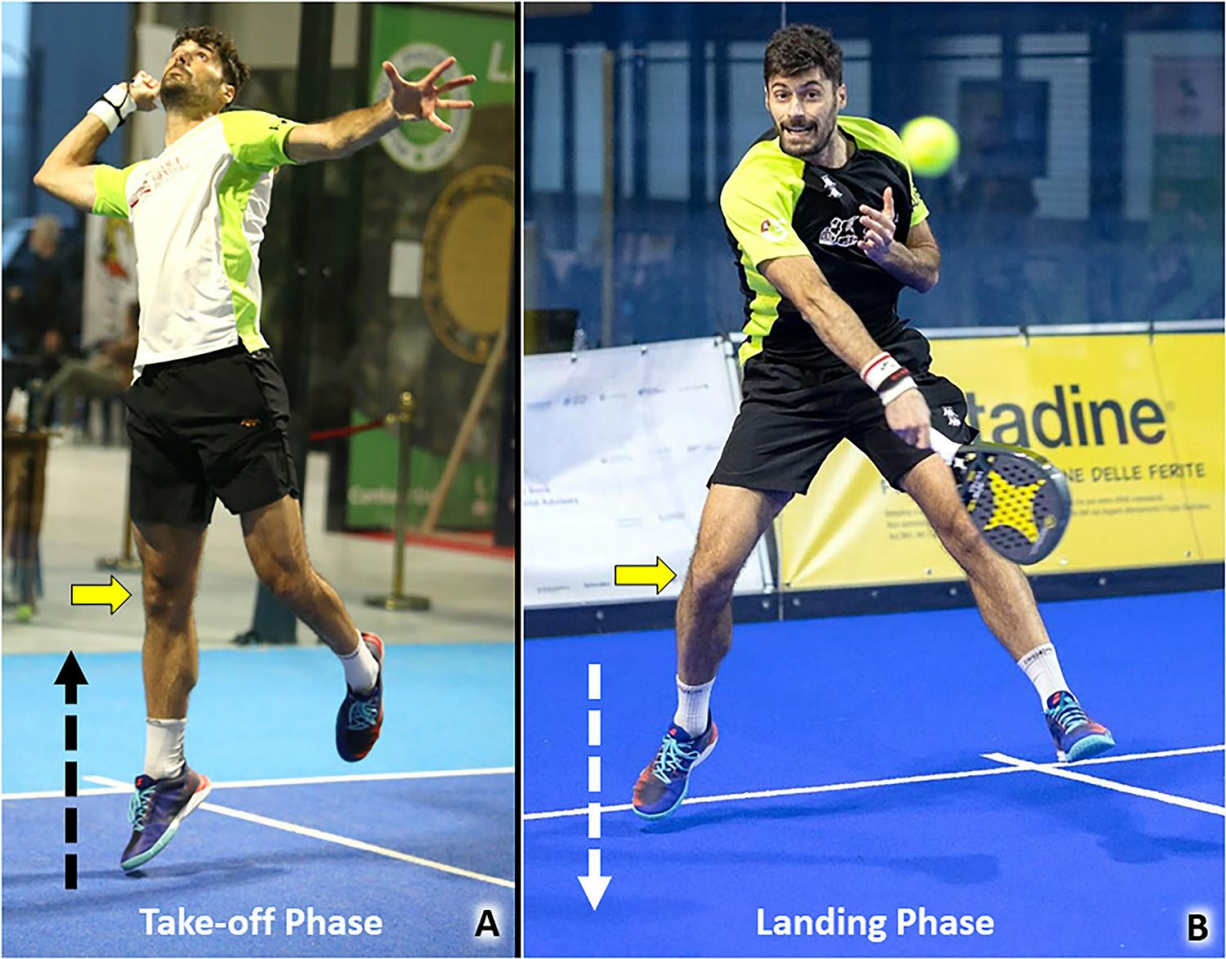
Powerful contraction of the triceps surae and extension of the knee *(yellow arrow)* are key biomechanical factors during the take-off phase of the jump *(black dotted arrow)* to reach the ball (**A**). Likewise, during the landing phase of the jump *(white dotted arrow)*, contraction of the gastrocnemius muscle with the knee slightly flexed *(yellow arrow)* allows soft braking protecting the ankle from an excessive mechanical overload (**B**)

Ankle plantarflexion coupled with a simultaneous and abrupt extension of the knee implies an active contraction and passive stretching of the gastrocnemius muscle. The aforementioned mix of elongation and contraction is a well-known biomechanical risk factor for myofascial/myotendinous injury of the medial gastrocnemius muscle (MGM) [48, 49]. The aforementioned biomechanical mechanism is very common during a padel match to rapidly change direction and to rise from the ground performing the so-called jump shots (Table 1). Specifically, during the take-off phase of a jump, an explosive contraction of the triceps surae is coupled with the knee extension to increase as much as possible the distance from the ground. Likewise, during the landing phase, the triceps surae contracts during passive dorsiflexion of the ankle and with the knee slightly flexed to optimize the braking and modulate the impact of the lower limb to the ground (Fig. 14). In this sense, in both the aforementioned phases the MGM actively contracts during its passive elongation – i.e., eccentric loads. Tagliafico et al., have reported 85 cases of MGM injuries in a cohort of 800 padel players [3]. The authors defined injury of the MGM as the second most common musculoskeletal disorder in this specific population after lateral elbow tendinopathy. Also known as tennis leg, this musculoskeletal injury can show an extremely variable histological/anatomical damage involving the muscle tissue of the medial gastrocnemius, its anterior aponeurosis, its free aponeurosis, and the posterior aponeurosis of the soleus muscle – i.e., the myo-aponeurotic complex of the triceps surae [48].

#### Clinical findings

Sudden pain in the calf and eventual feeling of “pop” are the most common symptoms complained by the player in the acute phase. Active/passive elongation of the gastrocnemius muscle elicits pain in the posterior compartment of the leg; and, the patient is usually unable to perform the single-leg heel raise test [50]. In the sub-acute phase, the calf tightens up and sometimes a local hematoma can be observed along the posteromedial surface of the leg extending to the medial compartment of the ankle. Interestingly, especially in patients with small-size injuries of the MGM or in cases of deeply located blood effusion; no superficial hematoma can be visually identified during the physical examination.

#### Sonographic findings

**Fig. 15:**
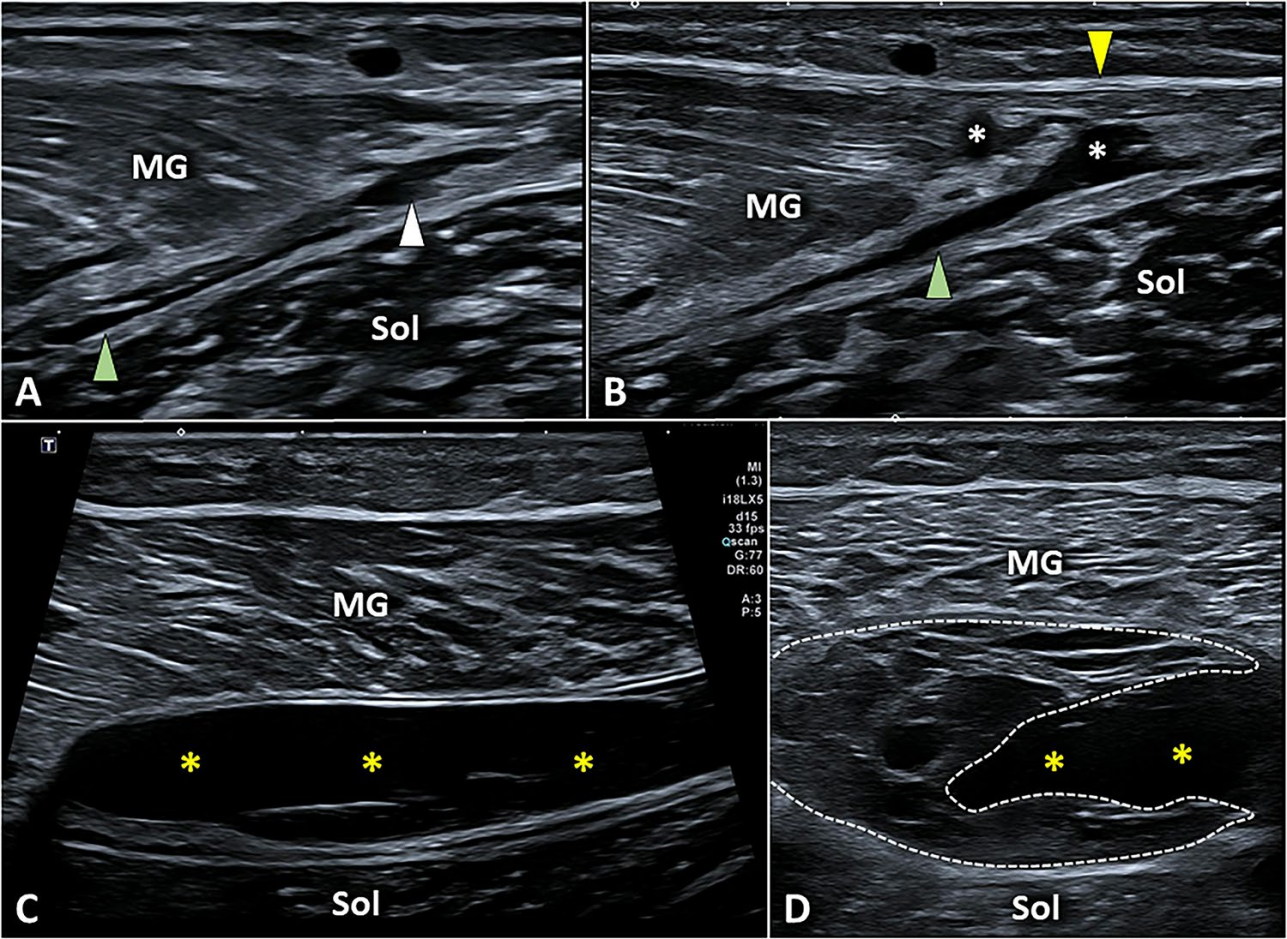
A small injury of the anterior aponeurosis *(white arrowhead)* of the medial gastrocnemius *(MG)* is enough to allow the blood to spill within the inter aponeurotic space *(green arrowhead)* of the triceps surae muscle (**A**). Instead, post-traumatic disruption of deep fibers of its free aponeurosis *(white asterisk)*, with sparing of superficial fibers *(yellow arrowhead)*, should be considered such as a tendon injury (**B**) with a worse functional prognosis compared to the previous myoaponeurotic lesion. In padel athletes with a large hematoma *(yellow asterisks)* in the inter aponeurotic space of the triceps surae muscle (**C**) ultrasound-guided drainage is paramount to avoid progression toward an irregular mass of granulation tissue *(white dotted line)* that “blocks” a correct healing process (**D**). *Sol* soleus muscle

As previously mentioned, sonographic signs of MGM injuries in padel players can be highly variable, involving the muscle tissue proper and/or its external connective scaffold. Post-traumatic discontinuity of its anterior aponeurosis implies a spilling of the hematoma within the intermuscular septum progressively dilatating the inter aponeurotic space between the MGM and the soleus muscle (Fig. 15) [48]. In padel players with a moderate to severe inter aponeurotic hematoma, the authors strongly suggest ultrasound-guided drainage coupled with rehabilitation treatments to avoid a progressive organization of the blood effusion with the development of (soft) granulation tissue [51]. Moreover, in patients with inter aponeurotic hematoma, and aponeurotic disruption involving more than 50% of the cross-sectional area of the muscle measured in a transverse plan; asynchronous movements between the MGM and soleus have been demonstrated during dynamic scanning—with plantarflexion and dorsiflexion of the ankle—with a longer return to play [51]. Lastly, disruption of its free aponeurosis can be histologically considered a tendinous injury with a worse prognosis and longer rehabilitation management for the return to play (Fig. 15) [48].

### Foot

#### Biomechanics

As previously mentioned for the PT and MGM, repetitive concentric and eccentric loads during the take-off and landing phases of the jump shots can be considered repetitive mechanical stresses on the Achilles tendon (AT) during a padel match (Fig. 14) [52]. The take-off phase requires a powerful plantar forefoot thrust with the knee extended to increase the distance from the ground as much as possible optimizing the so-called *elevation performance*. Likewise, during the landing phase, the AT acts as an elastic spring curbing the ankle dorsiflexion approaching the ground (Fig. 14). The latter biomechanical mechanism is paramount to avoid ankle sprains with violent dorsiflexion and loss of balance landing phase from the jump.

Moreover, the multiple rapid directional changes due to the irregular ball bouncing off the perimetral walls of the playing field may lead to extra friction between the AT and the surrounding elastic sleeve (paratenon) with a higher risk of peri tendinitis compared to tennis. Indeed, overuse syndromes of the gastrocnemius muscle lead to a repetitive overstretch of the crural fascia with thickening/inflammation of the underlying paratenon [53].

#### Clinical findings

##### Non-insertional Achilles tendinopathy

The most common clinical finding by inspecting the AT is the presence of a fusiform thickening usually located from 2 to 6 cm proximally to its calcaneal insertion. The squeeze test by pinching the tendon thickening with two fingers of the hand often reproduces the pain. Stiffness of the gastrocnemius-soleus complex during passive stretching is another frequent complaint by the athlete especially in chronic cases. Sometimes, by placing the hand over the AT and asking the patient for active movements of the ankle, a grinding sensation can be felt called crepitus [54]. The latter clinical sign may be related to the presence of local adhesions in the AT-paratenon gliding interface.

##### Insertional Achilles tendinopathy

The pain is mainly located at the attachment site of AT over the posterior aspect of the calcaneus. Active/passive elongation of the tendon can be used as a clinical test to increase the mechanical stress to the enthesis and reproduce the painful feeling. The presence of tenderness over the medial and lateral aspect of the insertional segment of AT may be related to the presence of deep retrocalcaneal bursitis [55]. Likewise, a painful pinching of the skin covering the distal portion of AT may be related to superficial retrocalcaneal bursitis. In some padel athletes, a bump can be identified at this level related to the presence of intra-tendinous calcification and/or bony spur of the calcaneus.

##### Achilles tendon rupture

The acute rupture of the AT usually onsets with severe pain and the inability to fully load the affected lower limb or walk on tiptoe [56]. The player presents impairment in walking losing the normal phases of rolling the foot on the ground. Physical examination may reveal a weakness in plantar flexion of the ankle, a positive Thompson’s test, and in some patients a soft spot along the course of the AT. Epidemiologically, the Achilles tendon rupture presents a bimodal distribution with a first peak commonly occurring in patients aged 25–40 years and a second peak in those older than 60 years [57]. Interestingly, in the first group of patients, the tendon rupture is often secondary to high-energy sport-relates trauma; instead, in the second group, low-energy trauma can be sufficient to trigger a tendon injury considering the pre-existing chronic Achilles tendinosis.

#### Sonographic findings

Achilles tendinopathy can be considered a generic medical definition that encompasses an extremely variable cluster of histological changes. Among many, midportion tendinosis, peri tendinitis, partial tear, enthesopathy, and superficial/deep retrocalcaneal bursitis are the most common. In padel players, very often several sono-histological changes coexist making the Achilles tendinopathy a real challenge for the clinician/surgeon.

##### Non-insertional Achilles tendinopathy

**Fig. 16:**
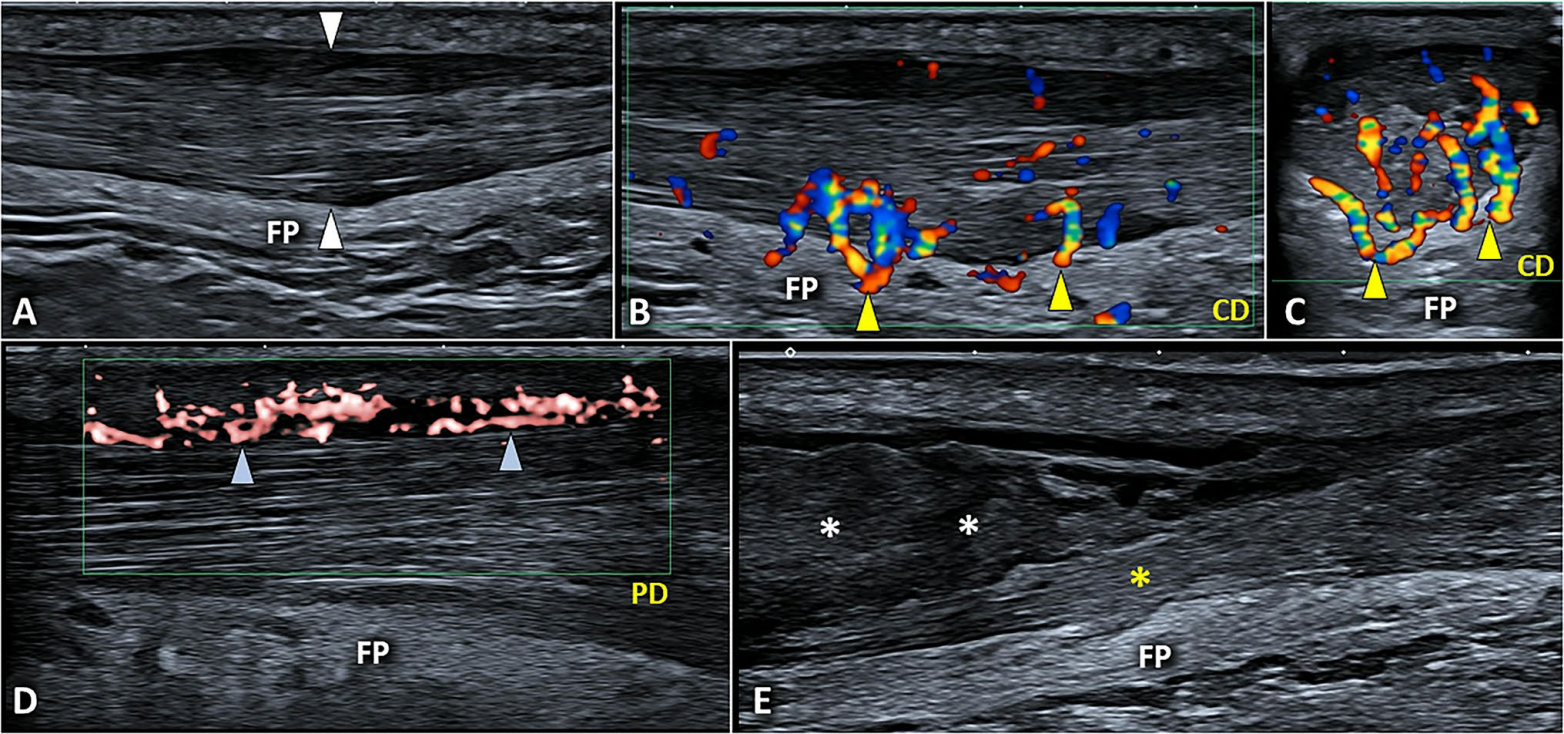
Fusiform hypoechoic thickening *(white arrowheads)* of the midportion of AT and its hypervascularization with neo-vessels *(yellow arrowheads)* originating from the underlying Kager fat pad *(FP)* can be considered the most common sonographic signs of non-insertional pathology (**A**–**C**). More rarely, isolated peri tendinitis may be identified with vascular signals *(blue arrowheads)* confined inside the thickened paratenon and a normal thickness/echotexture of the underlying AT (**D**). In players with suspected partial injury of the AT, ankle dorsiflexion may be performed to dynamically and selectively glide the disrupted tendon fibers *(white asterisks)* confirming the diagnosis (**E**). *CD* color doppler, *PD* power doppler

Hypoechoic thickening of the tendon is the most common sonographic finding in padel players with non-insertional Achilles tendinopathy (Fig. 16). Loss of the fibrillar pattern in the longitudinal plane, and a rounded shape in the transverse plane, are typical signs of focal tendinosis at this level. Color/power Doppler often shows neo-vessels originating from the underlying Kager fat pad and invading the degenerated segment of the AT (Fig. 16). Of note, neo-nerves are coupled with neo-vessels and seem to play a pivotal role in the genesis of tendon pain through a neurogenic inflammation mechanism [58]. Another common sonographic finding is the hypoechoic thickening of the paratenon, coupled with the midportion Achilles tendinosis or (more rarely) as a unique sonographic sign. Longitudinally the peri tendinitis simply appears as a hypoechoic band in between the subcutaneous tissue and the dorsal surface of the AT; instead, in a transverse plane it presents an inverted U-shape surrounding the dorsal, medial, and lateral aspects of the tendon [59]. Using high-sensitive color/power Doppler and accurately setting the pulse repetition frequency, hypervascularization surrounding the AT can be observed especially in players with acute peri tendinitis (Fig. 16).

Lastly, midportion Achilles tendinosis if not accurately managed may progress to mechanical disruption of tendon fibers – i.e., partial or complete tear. Proximal/distal stump usually presents a glove finger shape and, dynamic scanning can be promptly performed to “open the gap” optimizing its visibility [60]. Sometimes a linear hyperechoic fibrillar structure may be observed crossing the space in between the stumps of AT which represents the plantaris tendon [61].

##### Insertional Achilles tendinopathy

**Fig. 17:**
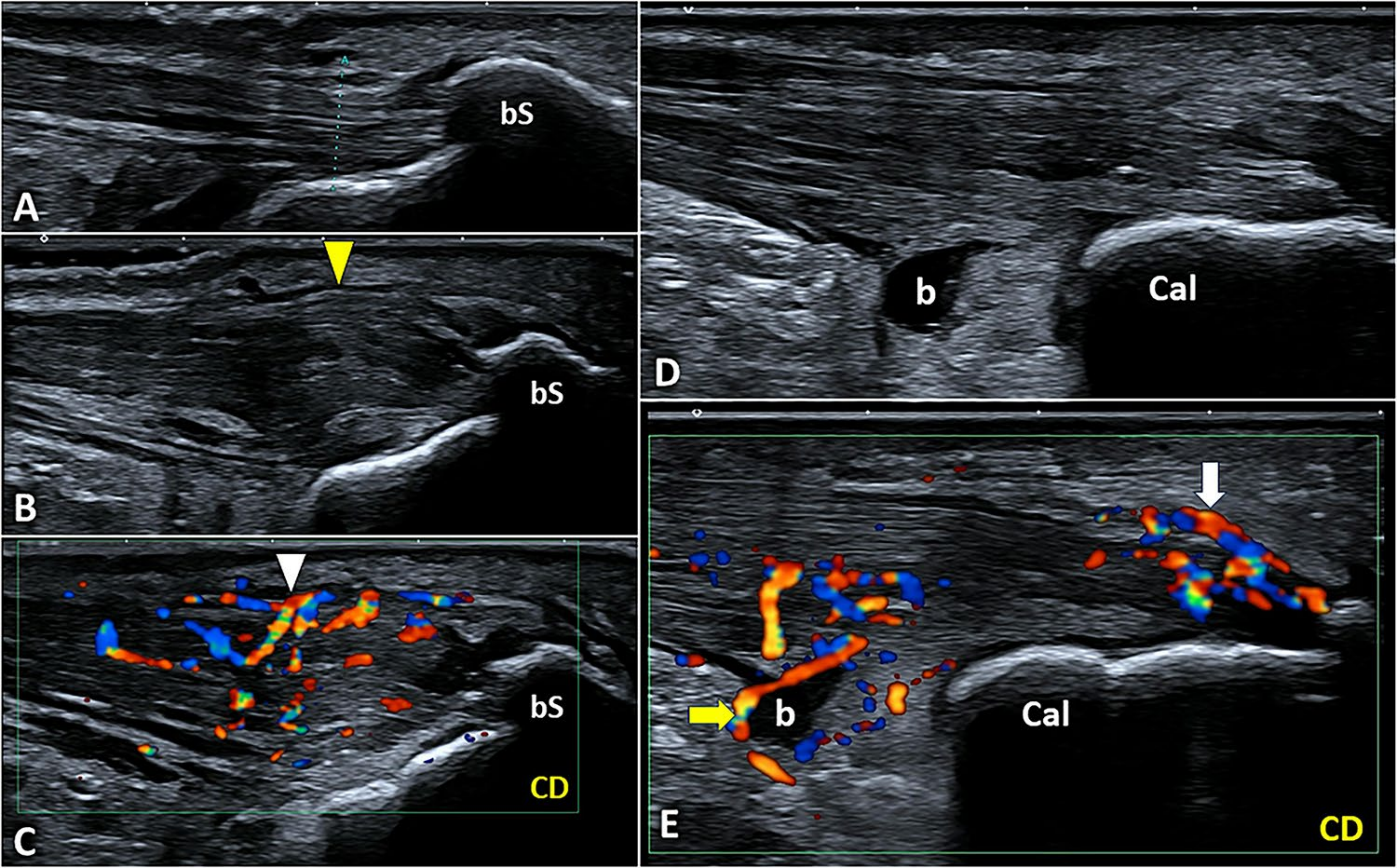
Comparative scanning (**A**, **B**) shows bilaterally the posterosuperior bony spur *(bS)* of calcaneus but, the hypoechoic thickening of the insertional portion of AT *(yellow arrowhead)* only on the painful side. The color/power Doppler *(CD)* confirms the presence of neo-vessels *(white arrowhead)* originating from the subcutaneous fat and penetrating the tendon tissue selectively on the painful heel (**C**). In another padel player with heel pain, the B-mode scan (**D**) showed effusion in the deep retrocalcaneal bursa *(b)*, and color Doppler *(CD)* confirmed its hypervascularization *(yellow arrow)* (**E**). Interestingly, hyperemia also involves the superficial retrocalcaneal bursa *(white arrow)* and the underlying tendon tissue (**E**). *Cal* calcaneus

Posterosuperior calcaneal bony spur is a very frequent sonographic finding in both symptomatic and asymptomatic padel players; instead, hypoechoic thickening of the insertional segment of AT, with coarse pattern and loss of fibrillar texture, seems to be a more specific sign of painful insertional tendinopathy (Fig. 17) [62]. Unlike the aforementioned midportion tendinopathy, color/power Doppler often reveals penetrating neo-vessels originating from the subcutaneous fat tissue rather than to the Kager fat pad in the insertional tendinopathy. Partial tendon injury, intra-tendinous calcifications, and enthesophyte of the tendon-bone interface may also be observed [63].

Effusion of the deep retrocalcaneal bursa is another common sonographic finding in padel players (Fig. 17) but, as the calcaneal spur, may be observed also in asymptomatic players. In this sense, the authors strongly suggest to perform always a comparative scanning of this anatomical region to better match to each other the clinical and ultrasonographic signs. Moreover, rather than the bursal effusion in M-mode, hyperemia of the synovial bursal walls in color/power Doppler seems to be a more specific sonographic sign of painful retrocalcaneal bursitis.

The superficial retrocalcaneal bursa is squeezed between the AT and superficial soft tissues so very rarely can be observed distended due to effusion. Usually, bursitis at this level shows hypoechoic thickening of the synovial lining with hypervascularization on color/power Doppler (Fig. 17) [64]. This anatomical structure, often overlooked, presents the highest density of nociceptors in patients with insertional Achilles tendinopathy compared to the tendon itself, the bone of calcaneus, and the deep retrocalcaneal bursa [65] In this sense, considering its pivotal role as a pain generator, some authors have also proposed the ultrasound-guided injection or debridement of the superficial bursal tissue [62].

## Conclusion

Musculoskeletal injuries are very common among padel players and a detailed knowledge of biomechanical features of padel-specific gestures is pivotal to performing a detailed sonographic assessment of the affected anatomical site. To the best of our knowledge, a head-to-head comparison between sport-specific movements and the corresponding sonographic findings is lacking in the pertinent literature, also considering the growth and diffusion recent of this sport. In this sense, the present research is intended as a ready-to-use guide to accurately perform the US examination in Padel players in daily practice.

## Abbreviations

US: Ultrasound
SASD: Subacromial-subdeltoid
ECRB: Extensor carpi radialis brevis
CET: Common extensor tendon
LE: Lateral epicondyle
CFPT: Common flexor-pronator tendon
PT: Patellar tendon
MGM: Medial gastrocnemius muscle
AT: Achilles tendon
